# Associations between meeting the Canadian 24-hour movement guidelines and physical, cognitive, social-emotional, and overall development in early childhood

**DOI:** 10.1186/s44167-022-00002-4

**Published:** 2022-09-01

**Authors:** Nicholas Kuzik, John C. Spence, Kevin Arkko, Clara-Jane Blye, Jenna Davie, Ria Duddridge, Tyler Ekeli, April English, Evelyn Etruw, Stephen Hunter, Carminda Goersch Lamboglia, Autumn Nesdoly, Madison Predy, Rebecca Rubuliak, Brendan Wohlers, Kelsey Wright, Valerie Carson

**Affiliations:** 1 Faculty of Kinesiology, Sport, and Recreation, University of Alberta, 1-151 Van Vliet Complex, Edmonton, AB T6G 2H9 Canada; 2 Faculty of Medicine and Dentistry, University of Alberta, Edmonton, AB Canada; 3 Faculty of Rehabilitation Medicine, University of Alberta, Edmonton, AB Canada

**Keywords:** Physical activity, Sedentary behaviour, Sleep, Preschool-aged children, 24-h guideline adherence

## Abstract

**Background:**

The importance of all movement behaviours (i.e., sleep, sedentary behaviour, and physical activity) for children's health has led to the creation of national and international 24-h movement behaviour guidelines for children. Few studies have examined the associations between guideline adherence and a broad array of health indicators in early childhood, and no study has done so with composite development scores for overall development. The objective of the present study was to examine associations for 24-h movement guideline adherence with physical, cognitive, social-emotional, and overall development indicators in a sample of 3–5-year-olds.

**Methods:**

Children (n = 95) were recruited for this cross-sectional study in Edmonton, Canada. Sleep, light-intensity physical activity, and moderate- to vigorous-intensity physical activity were measured with ActiGraph wGT3X-BT accelerometers. Screen time was measured via parental-report. Guideline recommendation adherence was categorized using the Canadian 24-h Movement Guidelines. Composite z-scores were created for physical (i.e., adiposity, growth, motor skills), cognitive (i.e., vocabulary, executive functions), social-emotional (i.e., self-regulation, social-emotional behaviours), and overall development. Linear regression models were conducted to examine associations between meeting different recommendation combinations (e.g., physical activity alone, combination of physical activity and sleep), and number of recommendations met (e.g., meeting only one of any of the recommendations) with each composite development outcome variable adjusted for relevant covariates.

**Results:**

Most children were 3–4 years old (77%) and males (69%). The physical activity guideline recommendation was the most frequently met single recommendation (94%), while the physical activity and sleep recommendations (80%) were the most frequently met combination of two recommendations. Further, 43% of children met all three recommendations. Meeting the sleep recommendation was positively associated with overall development (B: 0.29; 95% CI: 0.08–0.50), while meeting both the sleep and physical activity recommendations was positively associated with overall (B: 0.28; 95% CI: 0.10–0.46) and physical (B: 0.27; 95% CI: 0.03–0.51) development.

**Conclusions:**

Meeting sleep recommendations alone, as well as the combination of sleep and physical activity recommendations were associated with better physical and overall development in this sample. Future research should continue to examine a broad array of development outcomes using longitudinal study designs across early childhood.

**Supplementary Information:**

The online version contains supplementary material available at 10.1186/s44167-022-00002-4.

## Background

Independent associations between movement behaviours (i.e., physical activity, sedentary behaviour, and sleep) and health indicators for physical, cognitive, and social-emotional development have been observed in children of all ages [1–6]. In recent years, attention has been placed on all movement behaviours and their interactions with each other in a 24-h day. This new focus has led to the development of 24-h movement guidelines by several countries as well as the World Health Organization (specific to under 5 years of age) [7]. For instance, Canada was the first to release 24-h movement guidelines for school-age children and youth (aged 5 to 17 years) in 2016 and for early years children (aged 0 to 4 years) in 2017, which included age-specific physical activity, sedentary behaviour, and sleep recommendations [8, 9].

Prior to the release of the national and international 24-h movement guidelines, research examining the associations between integrated movement behaviours and health indicators was sparse [10, 11], especially for early childhood or the first 5 years of life. However, the new guidelines have resulted in an increase of studies examining all 24-h movement behaviours and various health indicators in early childhood [12]. This research has commonly used two approaches: compositional analyses or guideline adherence analyses [12]. The former is a technique used to overcome collinearity when examining data that is meaningfully interpreted as a proportion of a whole [13]. For instance, using compositional analyses [14] in a sample of 3- to 5-year-olds, we previously found a favourable trend for physical development when substituting 30 min of other movement behaviours for moderate- to vigorous-intensity physical activity (MVPA). Guideline adherence, the second approach, refers to meeting or not meeting public health guidelines. Using guideline adherence analyses allows for an understanding of the associations for thresholds (e.g., ≥ 60 min/day of MVPA) or ranges (e.g., 10–13 h/day of total sleep) of movement behaviours. According to a systematic review, meeting movement behaviour thresholds or ranges set by 24-h movement guidelines was beneficial for multiple indicators of physical, cognitive, and social-emotional development in early childhood [12].

Early childhood is one of the most critical and sensitive developmental periods in life, when children are susceptible to experiences that can be beneficial or deleterious to lifelong physical, cognitive, and social-emotional development [15–17]. Thus, a comprehensive approach is warranted to understand how 24-h movement guideline adherence impacts overall development. To date, most studies have focused on only one aspect of development (e.g., body mass index [BMI] z-score), with only one study of Australian children examining the association between meeting guidelines and health indicators for physical, cognitive, and social-emotional development [18]. Specifically, Hinkley et al. [18] found favourable longitudinal associations between certain recommendations within the guidelines and some indicators of physical and cognitive development, but null associations were observed with social-emotional development. However, no studies have examined the associations between guideline adherence and composite physical, cognitive, social-emotional, and overall development outcomes. Creating composite development scores [e.g., Early Childhood Development [19], Battelle Developmental Inventory [20]] can take into account the interconnectedness of children’s development. For instance, musculoskeletal growth leads to a body capable of increasingly complex fine (e.g., grasping) and gross (e.g., walking) motor skills, which further enhances growth (e.g., muscle development) through practice [21]; using an overall development score would simultaneously consider the contributions to development from growth and motor skills. The present study aimed to address these gaps by examining associations for adherence to 24-h movement guidelines with health indicators for composite physical, cognitive, social-emotional, and overall development in a sample of Canadian children aged 3–5-years. Further, considering the associations between physical, cognitive, and social-emotional development have previously been examined using compositional analyses in this sample [14], we sought to use the same data to compare findings with the guideline adherence approach, given these two approaches are fundamentally different.

## Methods

### Participants and procedures

Participants were children aged 3–5 years from the Parent–Child Movement Behaviours and Preschool Children’s Development study. Full details regarding participants and procedures have been previously published [14, 22, 23]. Briefly, parents with an eligible child or children were recruited either in person, via email, or social media from summer camps, classes, email lists, and social media platforms as part of a local division of a sport programming organization called Sportball. From July–November 2018, a total of 131 participants from Edmonton, Canada and the surrounding areas were recruited and consented to participate in this study. Of a possible 102 children attending summer camps, 60 children had parents provide consent to participate, though reasons for not participating were not tracked. Further, participation rates or reasons for non-participation were not measured from classes, emails, or social media due to logistical constraints. Ethics approval was received from the University of Alberta Research Ethics Board (Study ID: Pro00081175).

Data collection began with assessment of children’s motor skills at a University of Alberta sport and recreation centre in groups with 1–5 children. Following the motor development assessment, children wore an ActiGraph WGT3X-BT accelerometer 24 h/day for 7 days. When collecting the accelerometers from families’ homes or another location of their choice (n = 2), children completed cognitive development assessments on an iPad, while parents completed a questionnaire that included demographic information, screen time, and social-emotional developmental indicators. Additionally, children’s height and weight were objectively measured.

### Measures

#### Guideline adherence

Since participants were aged 3–5 years, the Canadian 24-Hour Movement Guidelines for the Early Years (aged 0–4 years) and for Children and Youth (aged 5–17 years) were used to assess guideline adherence [8, 9]. Surveillance recommendations from the guideline development processes were followed to determine adherence to specific physical activity, sedentary behaviour, and sleep recommendations.

##### Physical activity

Children aged 3–4 years were categorized as meeting the physical activity recommendations if on average they had at least 180 min/day of total physical activity (TPA) and at least 60 min/day of MVPA, while children aged 5 years needed at least 60 min/day of MVPA with no requirement for TPA. Physical activity was measured with the ActiGraph WGT3X-BT accelerometer, worn on the right hip, 24-h/day for 7 days, programmed at 30 Hz, and downloaded in normal-filtered 15-s epochs. TPA was classified as > 26 counts/15 s, and MVPA was classified as ≥ 420 counts/15 s [24]. Non-wear time was defined as ≥ 20 min of consecutive zeros in the accelerometer data [25]. A valid day was defined as at least 10 h of waking accelerometer wear time, and at least 2 valid days of accelerometer data were required to be included in the analysis [25].

##### Sedentary behaviour

Children aged 3–4 years were categorized as meeting the sedentary behaviour recommendation if on average they had no more than 1 h/day of screen time, while children aged 5 years met the recommendation if they had had no more than 2 h/day. The time per weekday and weekend day children spent viewing television, videos, or DVDs on a portable device, computer, or television were reported by parents in a questionnaire. Additionally, parents reported the weekend and weekday time children spent playing video/computer games on a variety of devices (e.g., cell phone, tablet, computer, consoles). The average total screen time/day was calculated by combining time spent playing video games and time spent viewing screens, then calculating the average daily duration based on weekend and weekday responses. These screen time items have demonstrated good test–retest reliability in a previous study [26].

##### Sleep

Children aged 3–4 years were categorized as meeting the sleep recommendation if on average they had 10–13 h/day of total sleep (i.e., daytime and nighttime sleep), while children aged 5 years required 9–11 h/day. Sleep was measured through visual inspection of low frequency extension filtered 15-s epoch ActiGraph data, with guidance through sleep log books and previously published visual inspection heuristics [27].

#### Development outcomes

Full details regarding the development outcome measures used in this study have been previously reported [14]. Briefly, outcome measures consisted of the domains of physical, cognitive, and social-emotional development.

##### Physical development

Physical development was operationalized as growth, adiposity, and motor skills. Height and weight of the children were measured with a stadiometer and scale, while the height of the biological parents was self-reported in the questionnaire. These measures were used to calculate the children’s current percent of expected adult height [28], and body mass index (BMI) z-scores according to the World Health Organization’s growth standards [29]. The Test of Gross Motor Development (TGMD)-2 was used to measure the children’s object control and locomotor skills, which are summed to calculate total motor skills [30]. Moderate to good reliability in scoring the TGMD-2 was observed in this sample (Intra-class correlation coefficient: 0.69–0.79) [14]. Further, the TGMD-2 has previously demonstrated moderate to strong validity (r: 0.49–0.63) and excellent reliability (0.99–0.81) [31].

##### Cognitive development

Cognitive development was operationalized as visual-spatial working memory, response inhibition, and language development. All cognitive development indicators were measured using the Early Years Toolbox, administered through an iPad [32]. Specifically, the Early Years Toolbox tasks included the Mr. Ant task (visual-spatial working memory), the Go/No-Go task (response inhibition), and the Expressive Vocabulary task (language development). Acceptable to good internal consistency reliability (Cronbach’s α: 0.78–0.90) in the Early Years Toolbox tasks was observed in this sample [14]. Further, the Early Years Toolbox has previously demonstrated moderate to strong validity (r: 0.40–0.60) for response inhibition, visual-spatial working memory, and expressive vocabulary, as well as good to excellent reliability (Cronbach’s α range: 0.84–0.95) for the response inhibition and expressive vocabulary assessments [32].

##### Social-emotional development

Social-emotional development was operationalized as externalizing, internalizing, prosocial behaviour, sociability, as well as cognitive, emotional, and behavioural self-regulation. All social-emotional development indicators were measured using the Child Self-Regulation and Behaviour Questionnaire (CSBQ), a component of the Early Years Toolbox that was printed and completed by a parent [32]. Good internal consistency reliability in all the CSBQ subscales was observed in this sample (Cronbach’s α: 0.75–0.82), except for internalizing (Cronbach’s α = 0.55) and prosocial behaviour (Cronbach’s α = 0.64) [14]. Further, the CSBQ has previously demonstrated moderate to very strong validity and acceptable to good reliability (Cronbach’s α: 0.74–0.89) [32].

#### Covariates

Covariates included in this study were assessed through the parental questionnaire and were selected based on a previous analysis in the sample [14]. Selected covariates included: child age (years), sex (groups: male, female), ethnicity (groups: Caucasian, non-Caucasian), number of siblings (groups: 0, 1, ≥ 2), home type (groups: one level [home has one floor or level], two levels [home has two levels or floors]), yard size (five options increasing in size from no yard to a large yard), household income (ten options increasing in size from less than $25,000 to more than $200,000), as well as parent age (years) and marital status (groups: married, not married). For descriptive purposes, yard size was collapsed into three categories and household income was collapsed into six categories due to low cell count for some response options. However, for analyses, yard size and household income were not collapsed and were treated as continuous variables.

### Analysis

Descriptive statistics were presented as categorical values for participant characteristics, including the frequency and percent for each category. The percent of participants meeting individual recommendations (i.e., physical activity, sedentary behaviour, and sleep), combinations of recommendations (e.g., physical activity and sedentary behaviour), and number of recommendations (i.e., 0–3 recommendations) within the guideline were calculated. Additionally, z-scores (centred to a mean of zero) were calculated for each developmental outcome variable (See “Development outcomes” section for a list of all variables). Then the mean z-score of each outcome variable was used to create composite scores for physical, cognitive, and social-emotional development. Total motor skills was not used in the calculation of the physical development composite score, since it is the combination of object control and locomotor skills and these components were already included. As well, BMI z-scores, internalizing, and externalizing were negatively scored before calculating the mean composite scores. Lastly, the composite physical, cognitive, and social-emotional scores were averaged (i.e., [physical + cognitive + social-emotional] / 3) to create an overall development composite score.

Bivariate regression models were built between each covariate and each developmental outcome variable (see Additional file 1: Table S1). When a covariate was significant (p < 0.05) for a specific outcome variable, that covariate was added to subsequent regression models examining associations with guideline adherence for that specific outcome variable. Next, multiple regression models were conducted examining associations between meeting different recommendation combinations (e.g., physical activity alone, combination of physical activity and sleep), and number of recommendations met (e.g., meeting only one of any of the recommendations) with each composite development outcome variable adjusted for relevant covariates. Cohen’s f^2^ values were calculated to determine the effect size of coefficients in multiple regression models, and defined as small f^2^ 0.02–0.14, medium f^2^ 0.15–0.34, and large f^2^ ≥ 0.35 [33].

Assumptions for regression analyses (i.e., linearity, normality, and equal variance of residuals, as well as identifying influential observations) were checked through visual inspection of residuals (i.e., residuals vs fitted values, Q-Q, square root of Standardized residuals vs. fitted values, and Cook’s Distance). All composite development models met the regression assumptions. The analyses for composite development outcome variables were also conducted for each individual development outcome in a supplementary analysis. Some Q-Q plots were not normally distributed for individual development outcomes, but the assumption of normality was met when participants with Cook’s D values > 4/n in multiple regression models were removed for expected adult height (n = 6–9; depending on the model), object motor skills (n = 3–6), and total motor skills (n = 3–6). The assumption of normality could not be met with transformations or removal of participants based on Cook’s D values for the variables externalizing and internalizing. Thus, categorical variables were created by splitting data by the median values (i.e., internalizing > 1 [56%], and externalizing > 2 [46%]), and logistic regressions were used instead of linear regression. All analyses were conducted in R version 3.6.1.

## Results

**Fig. 1:**
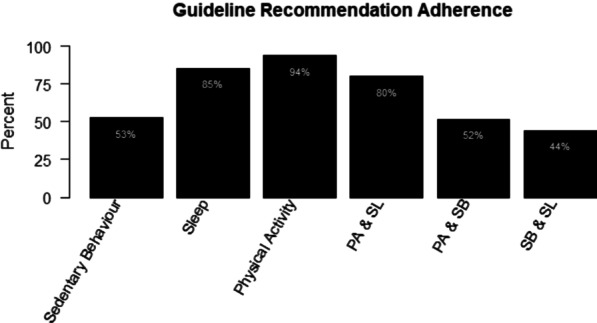
Guideline recommendation adherence as a percent, grouped by isolated recommendation adherence and combined recommendation adherence. *PA* physical activity recommendations, *SL* sleep recommendations, *SB* sedentary behaviour recommendations

**Fig. 2:**
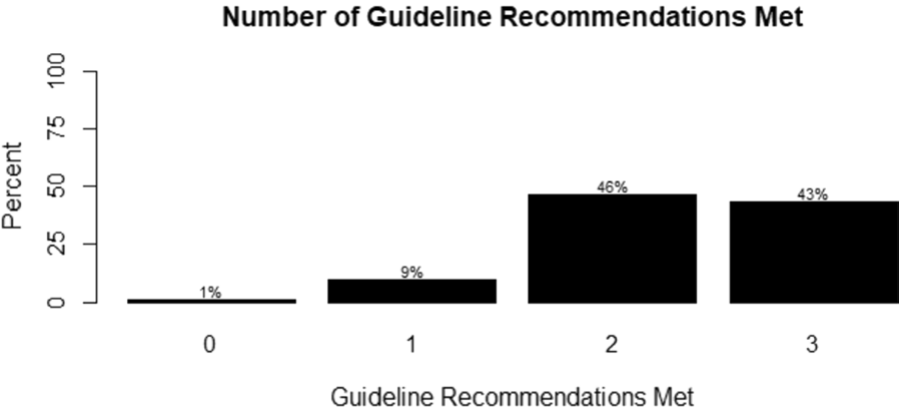
Number of guideline recommendations met as a percentage, ranging from not meeting any recommendations (0) to meeting all recommendations (3)

Of the 131 recruited children, 95 had movement behaviour data. The 95 participants in the analytical sample had all outcome variables except for response inhibition (n = 93) and all motor skill outcomes (n = 93). Thus, 93 participants had composite cognitive and physical development scores, while 91 participants had overall development scores. Most children were in the 3–4 year age group (77%) (See Table 1 for a full list of participant characteristics). On average, children accumulated 4.95 ± 0.60 (mean ± SD) hours/day of LPA, 1.76 ± 0.48 h/day of MVPA, 1.52 ± 1.05 h/day of screen time, and 10.93 ± 0.96 h/day of sleep. Additionally, on average, children had 6.06 ± 1.04 valid days of accelerometer data, and 12.82 ± 1.11 h/day of waking wear time. The physical activity recommendations were the most frequently met single recommendation (94% of sample), while the physical activity and sleep recommendations (80%) were the most frequently met combination of two recommendations (See Fig. 1). For those not meeting the sleep recommendations (15%), half of the participants were above the recommended range. Additionally, 43% of the sample met all three recommendations within the guidelines (See Fig. 2). Lastly, the distribution of guideline recommendation adherence for 3–4-year-olds was 91.78% for physical activity, 38.36% for screen time, and 87.67% for sleep; compared to 5-year-olds with 100.00% for physical activity, 72.73% for screen time, and 68.18% for sleep.

**Table 1:** Participant Characteristics

Covariate		Frequency (%)
Age Group (year)	3–4	73 (76.84)
	5	22 (23.16)
Sex	Male	66 (69.47)
	Female	29 (30.53)
Ethnicity	Caucasian	68 (71.58)
	Non-Caucasian	27 (28.42)
Siblings	None	15 (15.79)
	One	52 (54.74)
	Two or more	28 (29.47)
Parental relation to child	Mother	77 (81.05)
	Father	18 (18.95)
Marital status	Married	85 (89.47)
	Not married	10 (10.53)
Household income	≤ $100,000	9 (9.47)
	$100,001–$125,000	14 (14.74)
	$125,001–$150,000	16 (16.84)
	$150,010–$175,000	18 (18.95)
	$175,001–$200,000	14 (14.74)
	> $200,000	24 (25.26)
Home type	One level	37 (38.95)
	Two levels	58 (61.05)
Yard size	≤ Small yard	10 (10.53)
	Medium yard	66 (69.47)
	Large yard	19 (20.00)

Meeting the individual sleep recommendation (B = 0.29, 95%CI: 0.08, 0.50) and both the physical activity and sleep recommendations (B = 0.28, 95%CI: 0.10, 0.46) were associated with higher overall development, with Cohen’s f^2^ values of 0.09 and 0.11 (small effects), respectively (Table 2). Additionally, meeting both the physical activity and sleep recommendations were associated with a higher physical development (B = 0.27, 95%CI: 0.03, 0.51) composite score (Cohen’s f^2^ = 0.09; small effect) (Table 2). Several significant positive associations were also observed for individual development outcomes (See Additional file 1: Table S2). First, for meeting the sleep recommendations with object control skills (f^2^ = 0.09; small effect) and behavioural self-regulation (f^2^ = 0.10; small effect). Second, for meeting the combination of sleep and physical activity recommendations with total motor skills (f^2^ = 0.08; small effect), object control skills (f^2^ = 0.12; small effect), and expected adult height (f^2^ = 0.06; small effect). Lastly, for the number of recommendations met with response inhibition (f^2^ = 0.05; small effect).

**Table 2:** Associations between guideline recommendation adherence and development

Guideline recommendations	Overall development B (95%CI)	Physical development B (95%CI)	Cognitive development B (95%CI)	Social-emotional development B (95%CI)
Sedentary behaviour	− 0.07 (− 0.23, 0.09)	− 0.06 (− 0.28, 0.15)	0.13 (− 0.12, 0.37)	− 0.04 (− 0.32, 0.24)
Sleep	**0.29 (0.08, 0.50)**	0.24 (− 0.04, 0.52)	0.24 (− 0.10, 0.58)	0.22 (− 0.15, 0.59)
Physical activity	0.22 (− 0.12, 0.57)	0.10 (− 0.33, 0.53)	0.02 (− 0.52, 0.55)	− 0.02 (− 0.59, 0.56)
Physical activity + sleep	**0.28 (0.10, 0.46)**	**0.27 (0.03, 0.51)**	0.19 (− 0.11, 0.48)	0.17 (− 0.16, 0.51)
Physical activity + sedentary behaviour	− 0.07 (− 0.23, 0.09)	− 0.01 (− 0.23, 0.20)	0.09 (− 0.16, 0.34)	− 0.05 (− 0.34, 0.23)
Sedentary behaviour + sleep	0.04 (− 0.13, 0.20)	0.03 (− 0.19, 0.24)	0.18 (− 0.07, 0.42)	0.04 (− 0.25, 0.33)
Meeting all recommendations	0.03 (− 0.13, 0.20)	0.08 (− 0.14, 0.30)	0.14 (− 0.10, 0.39)	0.03 (− 0.26, 0.32)
Number of recommendations met	0.08 (− 0.04, 0.19)	0.05 (− 0.10, 0.20)	0.14 (− 0.04, 0.32)	0.04 (− 0.16, 0.24)

All analyses treated not meeting recommendations as the reference value. *B* unstandardized coefficient, *95%CI*  95% confidence intervals, Bolded values are significant at p < 0.05

## Discussion

The objective of this study was to examine associations for adherence to 24-h movement guidelines with physical, cognitive, social-emotional, and overall development composite scores in a sample of 3–5-year-olds. Meeting the sleep recommendation was positively associated with overall development. Meeting both the sleep and physical development recommendations was positively associated with overall and physical development. Small effect sizes were found for all significant relationships. Further, no associations were observed for sedentary behaviour recommendations.

Positive associations between adherence to 24-h movement guidelines and indicators of physical development were found in a recent longitudinal study [18]. Specifically, meeting the physical activity, sedentary behaviour, and sleep recommendations at age 3 to 5 years was associated with lower BMI z-scores at ages 9–11 years. In contrast, while the current study found associations for the physical development composite score, no associations were found specifically for BMI z-scores. Additionally, no associations were found between guideline adherence and BMI z-scores in a Canadian nationally representative cross-sectional sample of children aged 3–4 years [34] and a Swedish nationally representative longitudinal study of children 4 years of age, followed-up at 1 year [35]. However, the lack of associations in these studies when compared to Hinkley et al. [18] may support the argument that adiposity is accumulated over a longer time period, and movement behaviours need a longer exposure period before effects can be observed [34]. Additionally, studying adiposity cross-sectionally in preschool-aged children is difficult due to adiposity rebound [36], thus observed longitudinal associations could result from assessing adiposity at an age with more stability in this regard.

Only adiposity and growth indicators were examined in the previous guideline adherence and physical development studies [18, 34, 35], whereas the current study also included motor skills. Subsequently, in this sample meeting a combination of physical activity and sleep recommendations was associated with a 5 point higher object motor skills score and an 8 point higher total motor skills score. Therefore, motor skills along with expected adult height strongly contributed to the overall physical development finding. To better understand which aspects of physical development are most related to movement behaviour patterns, more studies examining guideline adherence that continue to use a broad array of physical development indicators are needed. Further, studies using longitudinal study designs are needed to examine any bidirectional associations between movement behaviours and development.

In this study, no significant associations were observed between guideline adherence and overall cognitive development, though the number of recommendations met was associated with higher response inhibition. McNeill et al. [37] also used the Early Years Toolbox [32] to measure cognitive development in children aged 3–5 years, followed up after one year. In agreement with the presented study, no significant associations were observed for guideline adherence at baseline and visual-spatial working memory at follow-up [37]. However, in contrast with the present study, McNeill and colleagues (2020) found no significant associations for guideline adherence at baseline and response inhibition at follow-up. Further, McNeill et al. [37] did find that meeting the physical activity recommendation was associated with higher cognitive shifting one year later. Unfortunately, the current study did not measure cognitive shifting, thus comparisons are not possible. Beyond the Early Years Toolbox, Hinkley et al. [18] found that meeting sleep guidelines for children aged 3–5 was associated with higher scores for reading, writing, spelling, numeracy, and language domains of academic achievement at ages 8–9 years. This could speak to the importance of early movement behaviour patterns for cognitive achievements (e.g., grades, academic awards) and cognitive abilities that develop later in childhood, such as cognitive shifting [38]. Future research should confirm longitudinal findings, while continuing to explore various cognitive achievement and ability assessments.

No significant associations were observed between guideline adherence and overall social-emotional development in this study, though meeting the sleep recommendations were associated with higher behavioural self-regulation. No studies could be found examining 24-h movement behaviour guideline adherence and behavioural self-regulation in early childhood, so comparisons are difficult. However, two longitudinal studies also found null associations for guideline adherence with behavioural and emotional problems (e.g., total problems, internalizing problems, externalizing problems) [18, 37]. This contrasts with a cross-sectional study of 3-year-old children that observed associations for meeting sedentary behaviour recommendations, and combinations including sedentary behaviour recommendations, with lower total problems, externalizing problems, and internalizing problems [39]. Future studies should explore potential mechanisms for the differences in study findings. Beyond behavioural and emotional problems, Cliff et al. [40] found that meeting recommendations for sleep, and combinations including sleep, were favourably associated with theory of mind and emotional comprehension. To better understand the myriad of indicators making up social-emotional development, future research should examine a broad spectrum of indicators in relation to movement behaviours.

Previous studies examining 24-h movement guidelines and development in a similar age group as this study have reported adherence as 19–94% for physical activity, 84–98% for sleep, 17–63% for sedentary behaviour, and 3–20% for all three [18, 34, 35, 37, 39, 40]. In the current study 94% of participants met the physical activity guidelines, which is similar to other studies (89%-94%) that also measured physical activity with a hip-worn ActiGraph accelerometer and used the same physical activity cut-points [18, 37, 40]. However, 94% is seemingly high compared to 19% when using a wrist-worn ActiGraph accelerometer and larger epochs [39], 31% when different physical activity cut-points are applied to the ActiGraph [35], and 62% when using the Actical accelerometer [34]. Additionally, in the current study participants had 43% adherence to all three guidelines, compared to the previous studies that showed 3–20% adherence [18, 34, 35, 37, 39, 40]. The sample examined in the current study may not be representative of the broader population based on their healthy movement behaviour patterns. In fact, two studies reported adherence to all three guideline recommendations as 3% in Canada [34] and 5% in Edmonton, Alberta [39]. Differences in findings could indicate this sample has poor generalizability or could demonstrate the issues when comparing estimates using different movement behaviour measurements. Thus, achieving consensus on the measurement of movement behaviours, especially accelerometer protocols (e.g., accelerometer brand, wear-site, cut-points), will allow for comparisons across future studies. As well, future research could examine relative-intensity accelerometer cut-points to better represent an individuals intensity of physical activity based on their fitness [41].

Meeting sleep recommendations or combinations of recommendations that include sleep are frequently reported to be favourable for children’s development [18, 37, 39, 40], including the results presented in the current study. Interestingly, when using the same data, no significant associations were observed in a compositional analysis between sleep and development outcomes, relative to the other movement behaviours [14]. Differences in results could exist since the previous study examined relationships linearly, whereas the current study examined guideline recommendation adherence based on a window of time. Some argue that the benefits of sleep for healthy development are not a linear relationship, instead benefits exist in a window of time (e.g., 10–13 h/day), or an inverted U shape [42]. In other words, both too little and too much sleep could have detrimental effects on development. Interestingly, of the participants not meeting sleep recommendations in this sample, half were above, and half were below the recommendations. Therefore, findings in the current study support the rationale of choosing a window of time, instead of a threshold, when creating sleep recommendations in the 24-Hour Movement Guidelines in Canada [5, 9]. Heterogeneity in findings may also be explained by the differences in analyses, as compositional analyses consider one movement behaviour in relation to all other movement behaviours, while the current study only considered combinations of movement behaviours.

In addition to comparisons with sleep, linear regressions from our previous compositional analysis also found null associations between physical activity (i.e., LPA and MVPA) with cognitive and social-emotional development. However, favourable associations were found between MVPA and physical development [14]. In contrast to the compositional findings, the current study did not find favourable associations between physical activity guideline adherence and physical development. It is important to note, 94% of this sample met the physical activity guidelines. Thus, in our sample the thresholds for physical activity guideline adherence are likely not sufficient to differentiate between those with higher MVPA and better physical development as seen in the previous compositional analyses. Further, our previous compositional analysis found favourable associations between accelerometer-measured stationary time and cognitive development [14]. Compared to the current null findings for screen time, this could indicate that this sample was engaged in non-screen based sedentary behaviours that have previously demonstrated favourable associations with cognitive development. For instance, Poitras et al. [6] found that reading to early years children was favourably associated with cognitive development. Therefore, future research should continue measuring a range of types of sedentary behaviours (e.g., screen time, stationary time, reading time) to better understand the mechanisms of these associations.

To our knowledge, no other studies examining 24-h movement behaviours have created composite scores to represent physical, cognitive, social-emotional, and overall development. This approach can be beneficial from a public health messaging perspective, since the use of these broader domains of development creates a more succinct finding, and subsequently more succinct messaging. Further, combining scores could be protective of acute performance issues on any one task (e.g., loss of attention) or questionnaire item (e.g., parent misunderstood). However, there are also limitations with creating a composite score. For instance, one outcome (e.g., motor skills) could be overly influential and thus over representative of physical development. The technique used in this study created equal weights for each outcome domain (i.e., physical, cognitive, and social-emotional), so the overall development composite score was equally represented by the three development domains. Differential weighting could have also been explored, such as regression weights and expert consensus weighting (e.g., important = multiply scores by 2, less important = multiply scores by 1) [43]. Future studies could compare different techniques for creating an overall development score, such as identifying a criterion measure for regression weighting and weighting based on an expert consensus process.

The main strength of this study was the inclusion of a variety of development outcomes, across the domains of physical, cognitive, and social-emotional development. Though the variety of developmental indicators measured could make replication difficult. Ideally, a future synthesis of the literature could help identify universal core measures to be included when assessing development, similar to efforts underway in the SUNRISE study [44]. Further, this study used device-based measures of physical activity and sleep, and the total screen time measure included screen viewing and video game playing on a variety of mediums (e.g., tablet, cell phones, television), instead of only the traditional television viewing. The study also had some limitations. For instance, the cross-sectional study design prevents causation from being inferred. Additionally, the small convenience sample was recruited from a sports-based program and may not be generalizable to the broader population. Though a previous review has suggested that > 2 accelerometer wear days (regardless of weekend or weekday) is sufficient in this age group [25], that was based on waking day wear protocols. Thus, the use of > 2 days could lack reliability in this sample since a waking wear day protocol was not used, and future research is needed to re-examine minimum wear time and valid days needed for reliable 24-h wear protocols. The low reliability scores in this sample for externalizing and prosocial behaviours may have limited the ability to detect significant associations for the social-emotional development composite score. Lastly, the multiple comparisons in our analyses may have increased the risk for Type I error.

## Conclusion

This study examined the associations for meeting 24-h guidelines with physical, cognitive, social-emotional, and overall development composite scores in 3–5-year-olds. Meeting sleep recommendations alone, as well as the combination of sleep and physical activity recommendations, were associated with better physical and overall development in this sample. Future research should continue examining a broad array of development outcomes using longitudinal study designs across the early childhood range.

## Supplementary Information

**Additional file 1: Table S1.** Model characteristics. **Table S2.** Associations between developmental indicators and guidelines recommendation adherence.

## Data Availability

The datasets used and/or analysed during the current study are available from the corresponding author on reasonable request, and pending approval from the University of Alberta Research Ethics Board.

## Abbreviations

BMI: Body mass index
TPA: Total physical activity
MVPA: Moderate- to vigorous-intensity physical activity
TGMD: Test of Gross Motor Development
PA: Physical activity
SB: Sedentary behaviour
SL: Sleep

## References

[1] Carson V, Hunter S, Kuzik N, Gray CE, Poitras VJ, Chaput J-P, et al. Systematic review of sedentary behaviour and health indicators in school-aged children and youth: an update. Appl Physiol Nutr Metab. 2016;41(6):S240–65. 27306432 10.1139/apnm-2015-0630

[2] Chaput J-P, Gray C, Poitras V, Carson V, Gruber R, Birken C, et al. Systematic review of the relationships between sleep duration and health indicators in school-aged children and youth. Appl Physiol Nutr Metab. 2016;41(6):S266–82. 27306433 10.1139/apnm-2015-0627

[3] Poitras VJ, Gray CE, Borghese MM, Carson V, Chaput J-P, Janssen I, et al. Systematic review of the relationships between objectively measured physical activity and health indicators in school-aged children and youth 1. Appl Physiol Nutr Metab. 2016;41(6):S197–239. 27306431 10.1139/apnm-2015-0663

[4] Carson V, Lee E-Y, Hewitt L, Jennings C, Hunter S, Kuzik N, et al. Systematic review of the relationships between physical activity and health indicators in the early years (0–4 years). BMC Public Health. 2017;17(5):854. 29219090 10.1186/s12889-017-4860-0 PMC5753397

[5] Chaput J-P, Gray CE, Poitras VJ, Carson V, Gruber R, Birken CS, et al. Systematic review of the relationships between sleep duration and health indicators in the early years (0–4 years). BMC Public Health. 2017;17(5):855. 29219078 10.1186/s12889-017-4850-2 PMC5773910

[6] Poitras VJ, Gray CE, Janssen X, Aubert S, Carson V, Faulkner G, et al. Systematic review of the relationships between sedentary behavior and health indicators in the early years (aged 0–4 years). BMC Public Health. 2017;17(5):868. 29219092 10.1186/s12889-017-4849-8 PMC5773886

[7] Tremblay MS. Introducing 24-hour movement guidelines for the early years: a new paradigm gaining momentum. J Phys Act Health. 2019;1(1):1–4. 10.1123/jpah.2019-0401 31711035

[8] Tremblay MS, Carson V, Chaput J-P, Connor Gorber S, Dinh T, Duggan M, et al. Canadian 24-hour movement guidelines for children and youth: an integration of physical activity, sedentary behaviour, and sleep. Appl Physiol Nutr Metab. 2016;41(6):S311–27. 27306437 10.1139/apnm-2016-0151

[9] Tremblay MS, Chaput J-P, Adamo KB, Aubert S, Barnes JD, Choquette L, et al. Canadian 24-hour movement guidelines for the early years (0–4 years): an integration of physical activity, sedentary behaviour, and sleep. BMC Public Health. 2017;17(5):874. 29219102 10.1186/s12889-017-4859-6 PMC5773896

[10] Saunders TJ, Gray CE, Poitras VJ, Chaput JP, Janssen I, Katzmarzyk PT, et al. Combinations of physical activity, sedentary behaviour and sleep: relationships with health indicators in school-aged children and youth. Appl Physiol Nutr Metab. 2016;41(6 Suppl 3):S283–93. 27306434 10.1139/apnm-2015-0626

[11] Kuzik N, Poitras VJ, Tremblay MS, Lee E-Y, Hunter S, Carson V. Systematic review of the relationships between combinations of movement behaviours and health indicators in the early years (0–4 years). BMC Public Health. 2017;17(5):849. 29219071 10.1186/s12889-017-4851-1 PMC5773877

[12] Rollo S, Antsygina O, Tremblay MS. The whole day matters: understanding 24-hour movement guideline adherence and relationships with health indicators across the lifespan. J Sport Health Sci. 2020;9(6):493–510. 32711156 10.1016/j.jshs.2020.07.004 PMC7749249

[13] Pawlowsky-Glahn V, Egozcue JJ, Tolosana-Delgado R. Modeling and analysis of compositional data. New Jersey: Wiley; 2015.

[14] Kuzik N, Naylor P-J, Spence JC, Carson V. Movement behaviours and physical, cognitive, and social-emotional development in preschool-aged children: cross-sectional associations using compositional analyses. PLoS ONE. 2020;15(8): e0237945. 32810172 10.1371/journal.pone.0237945 PMC7433874

[15] Nelson III CA, Zeanah CH, Fox NA. How early experience shapes human development: the case of psychosocial deprivation. Neural Plasticity. 2019. 10.1155/2019/1676285 PMC6350537 30774652

[16] Knudsen EI. Sensitive periods in the development of the brain and behavior. J Cogn Neurosci. 2004;16(8):1412–25. 15509387 10.1162/0898929042304796

[17] Heckman JJ. Skill formation and the economics of investing in disadvantaged children. Science. 2006;312(5782):1900–2. 16809525 10.1126/science.1128898

[18] Hinkley T, Timperio A, Watson A, Duckham RL, Okely AD, Cliff D, et al. Prospective associations with physiological, psychosocial and educational outcomes of meeting Australian 24-Hour Movement Guidelines for the Early Years. Int J Behav Nutr Phys Act. 2020;17(1):36. 32151254 10.1186/s12966-020-00935-6 PMC7063763

[19] (UNICEF) UNCsF. Multiple Indicator Cluster Surveys (MICS-6): Questionnaire for Children Under Five 2020. https://mics.unicef.org/tools.

[20] Newborg J, Company RP. Battelle developmental inventory: Riverside Pub.; 2005.

[21] Grioup WMGRS, de Onis M. Relationship between physical growth and motor development in the WHO Child Growth Standards. Acta Paediatr. 2006;95:96–101. 10.1111/j.1651-2227.2006.tb02380.x 16817683

[22] Kuzik N, Spence JC, Carson V. Machine learning sleep duration classification in preschoolers using waist-worn ActiGraphs. Sleep Med. 2021;78:141–8. 33429290 10.1016/j.sleep.2020.12.019

[23] Kuzik N, Naylor P-J, Spence JC, Carson V. Parent–child movement behaviors and bluetooth proximity in preschool-aged children. Meas Phys Educ Exerc Sci. 2021. 10.1080/1091367X.2021.1914051.

[24] Janssen X, Cliff DP, Reilly JJ, Hinkley T, Jones RA, Batterham M, et al. Predictive validity and classification accuracy of ActiGraph energy expenditure equations and cut-points in young children. PLoS ONE. 2013;8(11): e79124. 24244433 10.1371/journal.pone.0079124 PMC3823763

[25] Cliff DP, Reilly JJ, Okely AD. Methodological considerations in using accelerometers to assess habitual physical activity in children aged 0–5 years. J Sci Med Sport. 2009;12(5):557–67. 19147404 10.1016/j.jsams.2008.10.008

[26] Carson V, Hesketh KD, Rhodes RE, Rinaldi C, Rodgers W, Spence JC. Psychometric properties of a parental questionnaire for assessing correlates of toddlers’ physical activity and sedentary behavior. Meas Phys Educ Exerc Sci. 2017;21(4):190–200.

[27] Tudor-Locke C, Barreira TV, Schuna JM Jr, Mire EF, Katzmarzyk PT. Fully automated waist-worn accelerometer algorithm for detecting children’s sleep-period time separate from 24-h physical activity or sedentary behaviors. Appl Physiol Nutr Metab. 2014;39(1):53–7. 24383507 10.1139/apnm-2013-0173

[28] Luo ZC, Albertsson-Wikland K, Karlberg J. Target height as predicted by parental heights in a population-based study. Pediatr Res. 1998;44(4):563. 9773847 10.1203/00006450-199810000-00016

[29] World Health Organization Multicentre Growth Reference Study Group. WHO Child Growth Standards based on length/height, weight and age. Acta Paediatr. 2006;95(S450):76–85. 10.1111/j.1651-2227.2006.tb02378.x 16817681

[30] Ulrich D. Test of gross motor development (TGMD-2). Austin, TX: PRO-ED. Inc; 2000.

[31] Griffiths A, Toovey R, Morgan PE, Spittle AJ. Psychometric properties of gross motor assessment tools for children: a systematic review. BMJ Open. 2018;8(10): e021734. 10.1136/bmjopen-2018-021734 PMC6224743 30368446

[32] Howard SJ, Melhuish E. An early years toolbox for assessing early executive function, language, self-regulation, and social development: validity, reliability, and preliminary norms. J Psychoeduc Assess. 2017;35(3):255–75. 28503022 10.1177/0734282916633009 PMC5424850

[33] Cohen J. Statistical power analysis for the behavioral sciences. New York: Academic Press; 1969.

[34] Chaput JP, Colley RC, Aubert S, Carson V, Janssen I, Roberts KC, et al. Proportion of preschool-aged children meeting the Canadian 24-Hour Movement Guidelines and associations with adiposity: results from the Canadian Health Measures Survey. BMC Public Health. 2017;17(Suppl 5):829. 29219075 10.1186/s12889-017-4854-y PMC5773883

[35] Berglind D, Ljung R, Tynelius P, Brooke HL. Cross-sectional and prospective associations of meeting 24-h movement guidelines with overweight and obesity in preschool children. Pediatr Obes. 2018;13(7):442–9. 29385654 10.1111/ijpo.12265

[36] Rolland-Cachera MF, Deheeger M, Bellisle F, Sempé M, Guilloud-Bataille M, Patois E. Adiposity rebound in children: a simple indicator for predicting obesity. Am J Clin Nutr. 1984;39(1):129–35. 6691287 10.1093/ajcn/39.1.129

[37] McNeill J, Howard SJ, Vella SA, Cliff DP. Compliance with the 24-Hour movement guidelines for the early years: cross-sectional and longitudinal associations with executive function and psychosocial health in preschool children. J Sci Med Sport. 2020;23(9):846–53. 32360244 10.1016/j.jsams.2020.02.011

[38] Diamond A. Executive functions. Annu Rev Psychol. 2013;64:135–68. 23020641 10.1146/annurev-psych-113011-143750 PMC4084861

[39] Carson V, Ezeugwu VE, Tamana SK, Chikuma J, Lefebvre DL, Azad MB, et al. Associations between meeting the Canadian 24-hour movement guidelines for the early years and behavioral and emotional problems among 3-year-olds. J Sci Med Sport. 2019;22(7):797–802. 30655179 10.1016/j.jsams.2019.01.003

[40] Cliff DP, McNeill J, Vella SA, Howard SJ, Santos R, Batterham M, et al. Adherence to 24-hour movement guidelines for the early years and associations with social-cognitive development among Australian preschool children. BMC Public Health. 2017;17(5):857. 29219104 10.1186/s12889-017-4858-7 PMC5773906

[41] Ozemek C, Cochran HL, Strath SJ, Byun W, Kaminsky LA. Estimating relative intensity using individualized accelerometer cutpoints: the importance of fitness level. BMC Med Res Methodol. 2013;13(1):53. 23547769 10.1186/1471-2288-13-53 PMC3617038

[42] Knutson KL, Turek FW. The U-shaped association between sleep and health: the 2 peaks do not mean the same thing. Sleep. 2006;29(7):878–9. 16895253 10.1093/sleep/29.7.878

[43] Bobko P, Roth PL, Buster MA. The usefulness of unit weights in creating composite scores: a literature review, application to content validity, and meta-analysis. Organ Res Methods. 2007;10(4):689–709.

[44] Okely AD, Reilly JJ, Tremblay MS, Kariippanon KE, Draper CE, El Hamdouchi A, et al. Cross-sectional examination of 24-hour movement behaviours among 3- and 4-year-old children in urban and rural settings in low-income, middle-income and high-income countries: the SUNRISE study protocol. BMJ Open. 2021;11(10): e049267. 10.1136/bmjopen-2021-049267 PMC8547512 34697112

