# Supplementary material for: Associations between meeting the Canadian 24-hour movement guidelines and physical, cognitive, social-emotional, and overall development in early childhood
Source: J Act Sedentary Sleep Behav. 2022 Sep 1;1:2. doi: 10.1186/s44167-022-00002-4 (PMC11934447; doi:10.1186/s44167-022-00002-4)
Supplement: Supplementary file 1 — Additional file 1: Table S1. Model characteristics. Table S2. Associations between developmental indicators and guidelines recommendation adherence. [file 44167_2022_2_MOESM1_ESM.docx]

| **Table S1:** Model Characteristics | |
| --- | --- |
| **Outcome** | **Included Covariates** |
| Behavioural Self-Regulation | none |
| BMI z-scores | Home Type |
| Cognitive Development | Child Age (years) |
| Cognitive Self-Regulation | Parent Age (years) |
| Emotional Self-Regulation | Siblings |
| Expected Adult Height (%) | Child Age (years), Sex, Parent Age (years), Household Income |
| Externalizing | none |
| Internalizing | Ethnicity |
| Locomotor Skills | Child Age (years) |
| Object Motor Skills | Child Age (years) |
| Overall Development | Child Age (years), Sex, Parent Age (years) |
| Physical Development | Child Age (years), Household Income |
| Prosocial Behaviour | Sex, Siblings, Yard Size |
| Response Inhibition | Child Age (years), Sex |
| Sociability | Yard Size |
| Total Motor Skills | Child Age (years) |
| Vocabulary | Child Age (years), Parent Age (years), Marital Status |
| Working Memory | Child Age (years) |

| **Table S2:** Associations Between Developmental Indicators and Guidelines Recommendation Adherence | | | | | | | | |
| --- | --- | --- | --- | --- | --- | --- | --- | --- |
| **Developmental Indicator** | **PA** | **SL** | **SB** | **PA & SL** | **PA & SB** | **SB & SL** | **Meeting All** | **Meeting #** |
| **Expected Adult Height (%)** | 0.012  (-0.006, 0.031) | 0.007  (-0.005, 0.019)^⊕^ | -0.006  (-0.014, 0.003) | 0.011  (0.001, 0.022)* | -0.003  (-0.012, 0.006) | -0.000  (-0.009, 0.009) | 0.002  (-0.007, 0.011) | 0.001  (-0.006, 0.007) |
| **BMI z-scores** | 0.255  (-0.457, 0.967) | 0.101  (-0.365, 0.568) | 0.087  (-0.258, 0.432) | 0.132  (-0.283, 0.548) | 0.083  (-0.261, 0.428) | 0.084  (-0.276, 0.443) | 0.080  (-0.279, 0.440) | 0.107  (-0.143, 0.357) |
| **Object Control Skills** | 3.488  (-2.202, 9.179) | 5.058  (1.427, 8.689)* | -1.996  (-4.855, 0.864) | 5.188  (2.011, 8.366)* | -1.411  (-4.289, 1.467) | -0.285  (-3.168, 2.598) | 0.305  (-2.596, 3.206) | 0.916  (-1.113, 2.945) |
| **Locomotor Skills** | 1.853  (-5.105, 8.811) | 1.315  (-3.279, 5.908) | 0.477  (-2.993, 3.947) | 2.529  (-1.521, 6.579) | 0.954  (-2.515, 4.423) | 0.730  (-2.739, 4.198) | 1.220  (-2.263, 4.703) | 0.843  (-1.606, 3.293) |
| **Total Motor Skills** | 5.809  (-4.756, 16.373) | 6.413  (-0.468, 3.294) | -0.854  (-6.208, 4.500) | 7.856 (1.853, 13.859)* | 0.230  (-5.134, 5.593) | 1.049  (-4.292, 6.390) | 2.154  (-3.206, 7.514) | 2.170  (-1.580, 5.920) |
| **Response Inhibition** | 0.116  (-0.070, 0.302) | 0.040  (-0.078, 0.157) | 0.077  (-0.007, 0.161) | 0.063  (-0.037, 0.164) | 0.066  (-0.018, 0.151) | 0.075  (-0.009, 0.159) | 0.064  (-0.021, 0.150) | 0.067  (0.005, 0.129)* |
| **Working Memory** | -0.137  (-0.783, 0.509) | 0.226  (-0.198, 0.650) | 0.044  (-0.276, 0.363) | 0.067  (-0.312, 0.445) | -0.002  (-0.322, 0.318) | 0.119  (-0.200, 0.437) | 0.074  (-0.247, 0.395) | 0.069  (-0.157, 0.295) |
| **Vocabulary** | 1.043  (-3.560, 5.645) | 1.006  (-2.087, 4.100) | -1.046  (-3.350, 1.257) | 1.442  (-1.275, 4.159) | -1.129  (-3.430, 1.172) | -0.628  (-2.975, 1.719) | -0.721  (-3.078, 1.636) | -0.115  (-1.773, 1.544) |
| **Cognitive**  **Self-Regulation** | -0.199  (-0.704, 0.307) | 0.099  (-0.231, 0.430) | -0.142  (-0.391, 0.107) | 0.102  (-0.196, 0.400) | -0.156  (-0.404, 0.093) | -0.030  (-0.290, 0.231) | -0.045  (-0.306, 0.217) | -0.071  (-0.254, 0.111) |
| **Behavioural Self-Regulation** | -0.450  (-1.008, 0.108) | 0.528  (0.177, 0.880)* | 0.077  (-0.198, 0.353) | 0.284  (-0.042, 0.610) | 0.036  (-0.240, 0.313) | 0.112  (-0.170, 0.395) | 0.069  (-0.215, 0.354) | 0.141  (-0.057, 0.339) |
| **Emotional**  **Self-Regulation** | 0.052  (-0.620, 0.725) | 0.195  (-0.237, 0.627) | -0.013  (-0.343, 0.317) | 0.176  (-0.214, 0.566) | -0.057  (-0.389, 0.274) | 0.103  (-0.234, 0.440) | 0.059  (-0.282, 0.400) | 0.059  (-0.180, 0.297) |
| **Externalizing^A^** | 4.674  (0.716, 91.475) | 1.133  (0.384, 3.455) | 1.112  (0.494, 2.505) | 1.539  (0.579, 4.301) | 1.204  (0.535, 2.720) | 0.886  (0.382, 2.035) | 0.962  (0.414, 2.223) | 1.290  (0.715, 2.377) |
| **Internalizing^A^** | 0.277  (0.014, 1.852) | 0.496  (0.143, 1.521) | 1.145  (0.493, 2.661) | 0.432  (0.140, 1.200) | 1.065  (0.458, 2.470) | 0.738  (0.311, 1.737) | 0.682  (0.286, 1.611) | 0.767  (0.407, 1.402) |
| **Sociability** | 0.411  (-0.130, 0.952) | -0.070  (-0.427, 0.286) | -0.181  (-0.446, 0.085) | 0.080  (-0.243, 0.402) | -0.132  (-0.399, 0.136) | -0.182  (-0.453, 0.090) | -0.132  (-0.406, 0.143) | -0.063  (-0.257, 0.130) |
| **Prosocial Behaviour** | -0.004  (-0.460, 0.453) | 0.064  (-0.219, 0.347) | -0.156  (-0.371, 0.059) | 0.051  (-0.204, 0.306) | -0.148  (-0.365, 0.069) | -0.113  (-0.334, 0.108) | -0.105  (-0.329, 0.119) | -0.060  (-0.215, 0.094) |
| Values represent B (95%CI) except for **^A^** (internalizing and externalizing), where values represent odds ratio (95%CI); PA=Meeting physical activity guideline recommendation; SL=Meeting sleep guideline recommendation; SB=Meeting sedentary behaviour guideline recommendation; Meeting #=The number of guideline recommendations met as a continuous variable; *= p-value < 0.05; ⊕= Became positive when removing Cook’s D outliers (>4/n). | | | | | | | | |
